# A Systematic Review and Meta-Analysis Evaluating Antibiotic Prophylaxis in Dental Implants and Extraction Procedures

**DOI:** 10.3390/medicina54060095

**Published:** 2018-12-01

**Authors:** Amrik Singh Gill, Hana Morrissey, Ayesha Rahman

**Affiliations:** School of Pharmacy, University of Wolverhampton, Wolverhampton WV1 1LY, UK; a.s.gill2@wlv.ac.uk (A.S.G.); Hana.Morrissey@wlv.ac.uk (H.M.)

**Keywords:** antibiotic prophylaxis, microbial drug resistance, dental extraction, dental implants

## Abstract

*Background and objectives:* The use of antibiotic prophylaxis in extraction and implant dentistry is still controversial, with varying opinions regarding their necessity. The overuse of antibiotics has led to widespread antimicrobial resistance and the emergence of multi drug resistant strains of bacteria. The main aim of this work was to determine whether there is a genuine need for antibiotic prophylaxis in two common dental procedures; dental implants and tooth extractions. *Methods:* Electronic searches were conducted across databases such as Cochrane Register of Controlled Trials, the UK National Health Service, Centre for reviews, Science Direct, PubMed and the British Dental Journal to identify clinical trials of either dental implants or tooth extractions, whereby the independent variable was systemic prophylactic antibiotics used as part of treatment in order to prevent postoperative complications such as implant failure or infection. Primary outcomes of interest were implant failure, and postoperative infections which include systemic bacteraemia and localised infections. The secondary outcome of interest was adverse events due to antibiotics. The Critical Appraisal Skills Programme tool was used to assess the risk of bias, extract outcomes of interest and to identify studies for inclusion in the meta-analysis. *Results:* Seven randomised clinical trials (RCTs) were included in the final review comprising *n* = 1368 patients requiring either tooth extraction(s) or dental implant(s). No statistically significant evidence was found to support the routine use of prophylactic antibiotics in reducing the risk of implant failure (*p* = 0.09, RR 0.43; 95% CI 0.16–1.14) or post-operative complications (*p* = 0.47, RR: 0.74; 95% CI 0.34–1.65) under normal conditions. Approximately 33 patients undergoing dental implant surgery need to receive antibiotics in order to prevent one implant failure from occurring. *Conclusions:* There is little conclusive evidence to suggest the routine use of antibiotic prophylaxis for third molar extractive surgery in healthy young adults. There was no statistical evidence for adverse events experienced for antibiotics vs. placebo. Based on our analysis, even if financially feasible, clinicians must carefully consider the appropriate use of antibiotics in dental implants and extraction procedures due to the risk of allergic reactions and the development of microbial drug resistance.

## 1. Introduction

Since the accidental discovery of antibiotics by Sir Alexander Fleming of Scotland in 1929, they have been the greatest contribution to the 20th century world of therapeutics [1]. Initially the first systemic antibiotics (penicillin and sulphonamides) were reserved only for military use during World War II due to the expense and complicated manufacturing processes. As these processes were simplified, new formulations were developed, access to antibiotics increased and widespread use began [2]. Thereafter was a significant reduction in morbidity and mortality that were associated with previously life threatening diseases such as scarlet fever, pneumonia, meningitis and diphtheria [3].

### 1.1. Antimicrobial Prescribing Trends in Dentistry

Dental extractions are commonly performed by dentists for a wide variety of reasons including dental caries, impacted teeth, orthodontic and periodontal treatment and trauma [4]. A dental implant is a surgical component used to replace missing teeth [5] which interfaces with the skull or jaw bone in order to support a dental prosthesis such as a crown. Bacterial contamination during implant placement is thought to be responsible for early implant losses and infected dental implants are difficult to treat, and 2% eventually will be removed [6]. Antibiotics are not simply alternatives to dental interventions, but act as an adjunct to treatment. They may indicate when clinical signs of involvement are evident. Prophylactic antibiotic treatment is the use of antibiotics before, during or after therapeutic, diagnostic or surgical procedures with the aim of preventing infectious complications. This differs to therapeutic antibiotic treatment which aims to clear infection caused by a colonising micro-organism [7]. A common practice among dentists worldwide is prescribing prophylactic antimicrobials for procedures causing bleeding in the oral cavity [8]. The use of prophylactic antibiotics in dental implants and extractions is highly controversial, with varying opinions regarding their necessity [8,9,10,11].

Dentists prescribe approximately 10% of all common antibiotics [12]. In dentistry, the use and indications for systemic antibiotics are limited as most dental and periodontal diseases are usually best managed by operative interventions and oral hygiene measures [13,14]. According to the National Centre for Disease Control and Prevention, approximately one in three outpatient antibiotic prescriptions are deemed unnecessary [14].

Prophylactic treatment is often decided on the presumption of an infection happening, for example, *Staphylococci*, *Streptococci* and anaerobic rods are the most common causes of wound infection in oral surgery [15] and as a result, broad spectrum antibiotics are typically prescribed, amoxicillin being the most common choice of clinicians [16,17]. Interestingly, a study conducted by Anderson et al. (2000) concludes that General Practioners (GPs) are more likely to prescribe antibiotics, specifically broad-spectrum, for acute dental problems in comparison to dentists. Table 1 provides a summary of the most commonly prescribed antibiotics used in current dentistry [18].

Studies investigating prophylactic antibiotic prescribing carried out in developed countries indicate that dentists have better clinical knowledge of antibiotic prescribing [17] compared to studies conducted in developing countries which reported the misuse of prophylactic antibiotic prescribing [17,18,19,20]. In 2010 India, China and United States of America were the top global consumers of antibiotics.

### 1.2. Antibiotic Resistance

According to the World Health Organisation (WHO), antibiotics are the most misused of all medicines due to ease of access, being inexpensive, familiar and with generally good safety profiles. This has led to the growing problem of antimicrobial resistance (AMR) which is becoming a global threat that could cause an eventual loss of antibiotic efficacy [23]. The Global Antimicrobial surveillance (GLASS) programme runs by WHO revealed 500,000 people across 22 countries with suspected infections becoming antibiotic resistant with microorganisms such as *Escherichia coli*, *Klebsiella pneumoniae*, *Staphylococcus aureus*, *Streptococcus pneumoniae*, and *Salmonella* spp. showing high rates of antibiotic resistance [24]. The European Union (EU) fact sheet on AMR estimates that antibiotic resistance results in approximately 25,000 deaths per year and in excess of €1.5 billion in related healthcare costs and productivity losses leading to resistance against different classes of antibiotics discovered to date [3,25]. Alanis (2005) reports that infections caused by the new strains of antibiotic resistant bacteria are not only difficult to treat but require longer courses of antibiotics and more complex therapy [2].

The new EU ‘One health action plan against AMR’ primarily aims to reduce the emergence and spread of AMR, thereby preserving the efficacy of existing antimicrobial agents for use in both humans and animals. In addition, greater monitoring and surveillance, strengthening infection prevention and control measures, promoting the appropriate use of antimicrobial agents followed by developing new alternative treatments and increasing awareness and understanding of AMR among both public and healthcare professionals were advocated by the EU action plan [26]. WHO has published a global strategy for the containment of resistance. The guidelines identified 68 recommendations calling for governments and health systems to produce their local guidelines [25,27].

### 1.3. Clinical Guidelines

Infective endocarditis (IE) is a severe infection causing inflammation of the endocardium due to a range of infectious agents including *Staphylococci*, *Streptococci*, fungi and *Pseudomonas aerunginosa* [28] and has a high mortality rate. Since many dental procedures cause bacteraemia, this may lead to invasive endocarditis in susceptible individuals. Previously, various national and international guidelines recommended that prior to invasive orthodontic procedures; those individuals at heightened risk of developing IE should be administered prophylactic antibiotics [17]. In general, prophylactic antibiotics are only recommended in surgery for:

- Patients at risk of infectious endocarditis (except in non-surgical dental procedures)
- Immuno-compromised patients
- For prolonged and extensive surgical interventions
- Surgery in infected sites
- When large foreign materials are implanted

In 2008 the National Institute for Health and Clinical Excellence (NICE) published clinical guidelines [29,30,31] on antibiotic prophylaxis against infective endocarditis (IE), recommending that antibiotics for the purpose of preventing the development of IE should not be given to adults and children at risk of IE who are undergoing dental procedures. Prior to this, preventative antimicrobials were prescribed for prevention of IE for many decades. The evidence for this decision was that a consistent association between a patient having an interventional procedure and the risk of developing IE could not be found [28]. Therefore, it is on this basis that the clinical effectiveness of prophylactic antimicrobials is not considered to be proven [31,32,33]. These guidelines further suggest prophylactic antibiotics used against IE for dental procedures are not cost effective [29,30]. According to NICE regular tooth brushing almost certainly presents a greater risk of IE than a single dental procedure because of repetitive exposure to bacteraemia with oral flora [29].

Antibiotics are however appropriate for oral infections where there is evidence of spreading infection (swelling, lymph node involvement and cellulitis) or systemic involvement such as malaise and fever according to the Scottish Dental Clinical Effectiveness Programme [13].

The National Health Service (NHS) dentists are required to observe the guidance of NICE whilst prescribing. Clinicians who work privately may not have the same contractual obligation to follow this guidance. They would however require strong justification to their local clinical commissioning groups (CCGs) for choosing not to do so [30].

Implementing a change in clinical practice has own set of problems, even if the proposed changes are evidence based via national guidelines, because it not only involves studying new evidence but also abandoning the old evidence [31]. Cottingham (2012) reports that much needs to be done in order to improve the understanding of NICE guidelines among the dental profession as only 62% of dental trainers and 69.7% of dental trainees have read the CG No. 64 guideline and 55.7% trainers and 77.6% trainees applied it [32].

### 1.4. Study Aim

The study aim was designed using Process, Intervention, Comparator and Outcomes (PICO) guidelines. The purpose of this systematic review was to determine whether there is a genuine need for antibiotic prophylaxis in two common dental procedures; dental implants and tooth extractions, for which antibiotics are still currently being prescribed as part of therapy.

## 2. Methods

### 2.1. Search Strategy

Initial electronic searches were conducted using the Cochrane Register of Controlled Trials, the UK National Health Service (NHS) Centre for reviews, Science Direct, PubMed and the British Dental Journal to identify clinical trials of either dental implants or tooth extractions, whereby the independent variable was systemic prophylactic antibiotics used as part of treatment in order to prevent postoperative complications (implant failure, infection).

The following search terms were used in various combinations in all specified databases: ‘dentistry’, ‘prophylactic antibiotics’, ‘antibiotic prophylaxis’, ‘infection’, ‘extraction’, ‘third molar’, ‘bacteraemia’, ‘implant’, ‘antimicrobial’. Primary outcomes of interest were implant failure, postoperative infections (including systemic), bacteraemia localised infections and other post-surgical related complications of infectious nature (fever, swelling, trismus, pain, purulent discharge, alveolar osteitis. Secondary outcomes of interest were any adverse events due to antibiotics).

### 2.2. Study Criteria

The study was designed based on the PRISMA guidelines to produce systematic review and perform metanalysis. Potential studies identified in the initial search were required to meet inclusion criteria; clinical randomised control trials investigating dental implant or tooth extraction for any indication using prophylactic antibiotics as part of treatment to prevent postoperative complications such as implant failure or infection. Clinical trials were also required to be published in English and from 2000 until 2013. Studies which did not contain a control group and were not randomised were also excluded from this review (Table 2). A double-blind RCT is of significant importance to eliminate the Hawthorn effect where patients may report fewer or more adverse events depending on personal beliefs or interpretation of the medication used [33].

### 2.3. Quality Assessment and Risk of Bias

Each study was critically appraised using the critical appraisal skills programme (CASP) tool checklist for clinical trials. To assess for risk of bias the RCTs were checked against four main quality criteria by the recommendations of the Cochrane Handbook for Systematic Reviews of Interventions Version 5.1.0 (study details for each criterion can be found in Appendix A and Appendix B):

1. Patient blinding
2. Assessor blinding
3. Allocation concealment
4. Participant compliance with follow-up

## 3. Results

**Figure 1:**
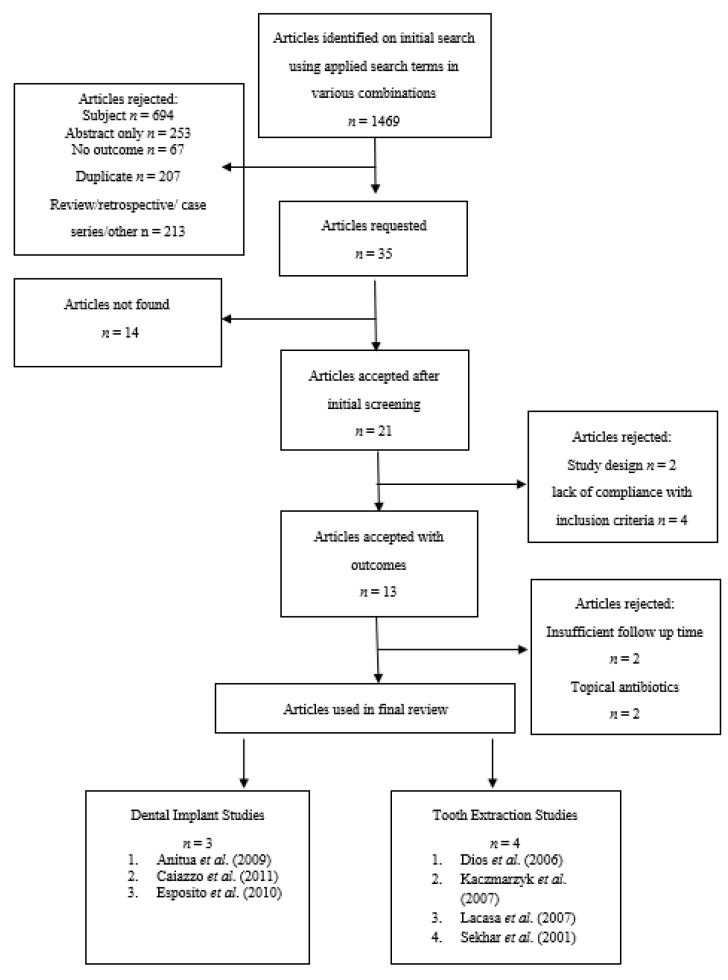
Flowchart showing the article selection process.

A total of 1469 articles were identified by the electronic searches conducted on the specified databases. Titles and abstracts were analysed for relevancy to this work resulting in 1434 articles being irrelevant and subsequently rejected. Thirty-five full text articles were reviewed, however 14 were irretrievable and 8 had a lack of compliance with inclusion criteria or inappropriate interventions and therefore rejected. Thirteen full text articles then underwent detailed analysis resulting in 6 further studies being excluded from this review. Seven randomised clinical trials (RCTs) were included in the final review comprising of a total of 1368 patients (657 extraction patients and 711 implant patients) requiring either tooth extraction(s) or dental implant(s). All included studies were published in English and complied with the inclusion criteria. All of these studies compared at least one type of antibiotic regimen against placebo in patients undergoing either dental extraction or implant placement (Figure 1).

Each of the randomised clinical trials used in this review were then categorised according to the level of bias as determined by the above specified criteria (Table 2 and Table 3).

Sekhar et al. (2001) was the only extraction study where multiple extractions per patient were allowed [12]. All participants were ≥18 years of age with various form of edentulism, however only one clinical implants study [34] included patients that required a single implant supported crown. The greatest difference in ratio of males to females in any arm of any study was seen in the Kaczmarzyk et al. (2007) placebo group [35]. Implant studies have a greater mean age than extraction studies. No study included elderly, young children or immune compromised patients (Table 4).

### 3.1. Extraction Studies

Four of the seven studies involved prophylactic antibiotics for dental extraction. All four extraction studies were multi-arm randomised control trials comprising of a total of 657 patients requiring single/multiple dental extractions (835 extractions) for various indications (impacted wisdom teeth, abscess etc). Three studies were conducted in Europe [34,35,36], whilst the fourth was conducted in India [12]. All extraction studies involved patients being treated at referral centres by oral surgery specialists rather than general dental practitioners. All extractions studies used local anaesthesia to perform dental extractions and each study contained compared at least one antibiotic regimen against placebo. Interestingly, the most common indications for dental extraction are caries or periodontal disease, yet no trials were identified which assessed the effect of prophylactic antibiotics in patients requiring dental extraction for these indications (Table 4).

#### 3.1.1. Dios et al. (2006) Trial

The authors performed microbiological analysis on post-operative bacteraemia present in blood cultures as an outcome measure to determine the effectiveness of prophylactic antibiotics in dental extraction. Dios et al. (2006) found *Streptococcus* spp. were the most commonly identified bacteria in all groups ranging from 44% to 68% with the lowest percentage being detected from the amoxicillin group (*p* < 0.0001). Amoxicillin and moxifloxacin prophylaxis showed high efficacies (*p* < 0.001 and *p* < 0.05 respectively) in reducing prevalence and duration of bacteraemia following dental extraction. Clindamycin prophylaxis was seen to be non-effective (*p* < 0.9). The results of the study therefore implicate that amoxicillin and moxifloxacin would be highly likely to reduce post-operate infections following dental extraction [36].

#### 3.1.2. Lacasa et al. (2007) Trial

The authors conducted a phase III comparative study evaluating the efficacy of two schedules of a sustained release amoxicillin/clavulanate preparation in order to reduce infection after third molar surgery. A total of 225 patients were randomised equally into three groups: placebo, prophylaxis using single pre-op dose of amoxicillin/clavulanate 2000/125 mg, and a pre-emptive therapy group given a matching placebo dose (2000/125 mg) pre-op followed by amoxicillin /clavulanate 2000/125 mg twice daily for 5 days. A statistically significant higher rate of infection was seen amongst the placebo group: 16% (12/75) vs. single dose prophylaxis: 5.3% (4/75) vs. 5-day pre-emptive therapy: 2.7% (2/72) (*p* = 0.006). A linear correlation was found between the length of procedure and rate of incidence (*p* < 0.027) probably due to the length of exposure associated with more lengthy and complex bone removal procedures (ostectomy). Both therapeutic and prophylactic regimes vs. placebo had achieved greater reduction of pain postoperatively (*p* = 0.0001). However, prophylaxis was seen to be more beneficial in cases where ostectomy is not performed [34]. Overall results favoured the use of pre-emptive antibiotic therapy to reduce the rate of subsequent infection in patients subjected to ostectomy and a single prophylactic dose to be useful in simpler extraction procedures. Out of 8 planned outcomes that were listed, only one was explained fully whilst pain was reported as a mean for each arm of the trial without estimate variance [37].

#### 3.1.3. Kaczmarzyk et al. (2007) Trial

This study involved 86 patients to evaluate the efficacy of a single and multi-dose clindamycin 5-day therapy to prevent inflammatory complications after third molar extractive surgery requiring bone removal. Clindamycin was chosen as it exerts strong antimicrobial action towards isolated strains from odontic infections as well as reaching high tissue concentrations. The only statistically significant result for any of the outcome measures (trismus, facial swelling, body temperature, pain, alveolar osteitis and lymphadenopathy) was a variation in body temperature was reported on the 7th day post-op (*p* = 0.03, Kruskal–Wallis rank test). All other outcome measure results in the study were *p* > 0.05, indicating a lack of statistical significance regarding efficacy in prophylaxis and pre-emptive therapy in any examined group. Results do not support the use of prophylactic antibiotics using clindamycin for preventing inflammatory complications in those requiring third molar extraction with bone removal under normal conditions [35].

#### 3.1.4. Sekhar et al. (2001) Trial

Sekhar et al. (2001) (high risk of bias) tested the efficacy of two dosing regimens of prophylactic antibiotics during removal of impacted lower third molars using 151 participants (Table 5). They used random allocation into three groups: placebo vs. prophylactic antibiotics pre-op vs. antibiotic treatment post-op for 5 days. Metronidazole was the antibiotic of choice but was not justified. Pain score, swelling and wound state were all assessed on day 2 and 6 postoperatively. Results showed no significant differences in the outcome between the three groups (*p* = 0.09). Between individual variables assessed (swelling, pain, wound discharge), the degree of swelling was significantly less in the 5-day antibiotic post-op group (*p* = 0.03). The study concludes that in this case, results failed to show advantage in any group. Prophylactic antibiotics did not reduce morbidity after the removal of impact third molars [12].

### 3.2. Dental Implant Studies

Three implant studies were included in the final review comprising of 711 patients (1225 implants). All three implant studies were randomised and double-blinded. Two studies were multicentre parallel studies [6,15] conducted in Italy whilst the third study [31] was conducted in Spain. All three multicentre trials were conducted in private dental practices. Only one trial was supported by the implant manufacturer [31,34]. One clinical study [6] used placebo and antibiotics which were donated from a generic drug manufacturing company (Table 6).

#### 3.2.1. Esposito et al. (2010)

Esposito et al. (2010) (low risk of bias) compared 2 g amoxicillin 1 h preoperatively with identical placebo tablets using 506 patients. Outcome measures of interest were prosthesis/implant failure, postoperative complications and adverse events. Ten participants experienced prosthesis failure in the placebo group in comparison vs. 4 in the antibiotic group. Severn implant failures occurred in the antibiotic group vs. 13 in the placebo group. The difference at patient level was not statistically significant (*p* = 0.083). The placebo group had twice the rate of infection vs. the antibiotic group (*n* = 8 vs. *n* = 4 respectively). Immediate post-extractive implants were more likely to fail in comparison to delayed implants (9% vs. 2% respectively). Although trends clearly favoured the antibiotic group, no statistically significant differences were observed for outcome measures and no adverse events were reported. The authors conclude that sample size was insufficient to show a statistically significant difference [6].

#### 3.2.2. Anitua et al. (2009)

Anitua et al. (2009) (low risk of bias) compared 2 g of amoxicillin 1 h preoperatively with identical placebo tablets when placing single implants in bone types II & III. The characteristics of saprophytic flora were also examined in all patients. A total of 105 patients were recruited (52 in antibiotic group and 53 in placebo group). The duration of follow up was 3 months after placement. In each group two participant experienced implant failures and 6 experienced postoperative infections. No statistically significant differences were found between groups for post-operative infection (*p* = 0.960). The authors found that the use of amoxicillin did not modify the natural saprophytic flora (*p* = 0.362). No adverse events were reported [34].

Overall, trends favour the use of antibiotics in implant, but results are not statistically significant in order to support the use of prophylactic antibiotics in single implant placement for any of the outcome measures.

#### 3.2.3. Caiazzo et al. (2011)

This study (high risk of bias) compared 4 interventions (*n* = 25 for each group): single dose 2 g amoxicillin 1-h pre-op vs. 2 g amoxicillin 1-h pre-op + 1 g twice daily for 7 days vs. 1 g amoxicillin post-op twice daily for 7 days vs. no antibiotic. The duration of follow up was 3 months after placement. No patients dropped out at any time. Two implant failures occurred in the placebo group vs. no failures in any of the 3 antibiotic groups (*n* = 75 patients). No statistically significant differences were observed for between groups (*p* > 0.05). No postoperative complications were reported in any group at weeks 1, 2, 4 and 8. No adverse events had been reported. Overall the authors concluded the lack of statistically significant evidence was perhaps due to the limited number of samples but still believe that implant placement may be one of the limited oral surgical procedures requiring routine antimicrobial prophylaxis [15].

### 3.3. Implant Failure

Data was pooled using REVMAN 5.0 software (Appendix A). Overall, results show more than twice the number of implant failures occurred in the placebo/no antibiotic group (4.8%) vs. antibiotic group (1.8%); RR 0.43; 95% CI 0.16 to 1.14.

The forest plot is a graphical representation of effect estimates and confidence intervals for each study using risk ratio (RR) and % weight as representation of event data. The blue box corresponds to the risk ratio point estimate and the % weight of each study is represented by the size of the box. Esposito et al. (2010) contributed the largest weighting (66%) for this particular outcome measure and is therefore represented by the largest blue box. Each horizontal line passing through a blue box depicts the 95% confidence interval (CI) range of intervention effects compatible with the study’s result. This indicates whether each effect was individually statistically significant for that particular study. The line of no effect is seen passing vertically through 1 (when using RR). The overall combined data (overall effect estimate) is graphically represented on the forest plot by a black diamond box. This provides a meta-analytic summary of all data for an outcome to provide the best possible estimate of the effect of the intervention with confidence interval. The height of the black box represents the RR (0.64), whilst the width represents the 95% CI (0.43; 0.16–1.14)

**Figure 2:**
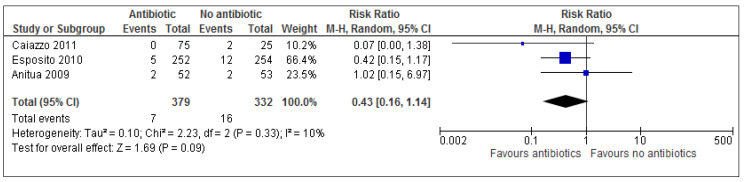
A forest plot of comparison showing antibiotics vs. placebo/no antibiotics for implant failure.

A risk ratio describes the multiplication of the risk which occurs due to experimental (antibiotic) intervention. Results show a risk ratio of 0.43 (95% CI 0.16–1.14), implying that antibiotics probably reduce the risk of implant failure by 57% (100 × (1 − RR)%) [35] based on these 711 patients under normal conditions. This is also known as the relative risk reduction. However according to the results of this review, prophylactic antibiotics were not statistically beneficial in those undergoing implant surgeries since *p* value (*p* = 0.09) for overall effect is greater than 0.05. This can also be seen in the forest plot (Figure 2) as each individual 95% CI passes through the line of no effect and the overall effect black box is in contact with the line of no effect. According to the Cochrane guidelines for systematic reviews of interventions 5.1.0, small study effects are difficult to identify with less than 10 studies and so a funnel plot may not be useful in this instance.

### 3.4. Adverse Events

**Figure 3:**
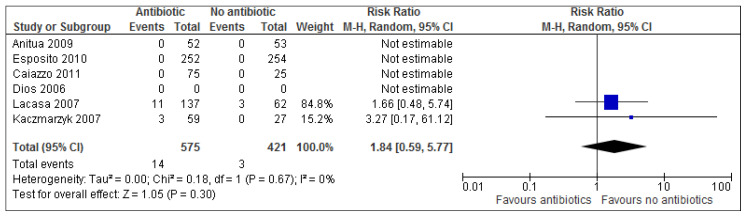
A forest plot of comparison showing antibiotics vs. placebo/no antibiotics for adverse events.

Adverse events were reported in only 2 out of 7 studies [32,34], of which only one study saw adverse events occur in the placebo/ no antibiotic group. Overall results (Figure 3) show that there is no statistical significance for adverse events (*p* = 0.30). The risk ratio of 1.84 (95% CI 0.59 to 5.77) implies (based on these results) that a patient is 1.84 times more likely to experience adverse events with treatment than adverse events without treatment. Alternatively, this can also be expressed as: 100 × (RR − 1)% = 100 × (1.84 − 1) = 100 × (0.84) = 84%. Treatment increases the risk of adverse events by 84% in the antibiotic group. Calculating the numbers needed to treat (NNT) helps understand the impact of an intervention on an outcome. Based on the results of this review, evidence suggests that approximately 33 patients undergoing dental implant surgery need to receive antibiotics in order to prevent one implant failure from occurring (Figure 2 and Table 7).

**Figure 4:**
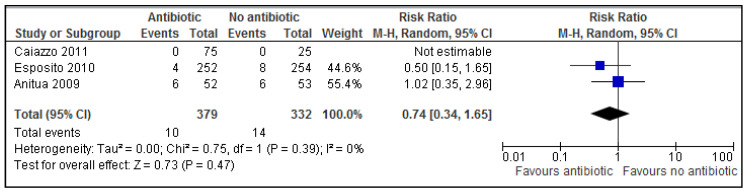
A forest plot of comparison showing antibiotics vs. placebo/no antibiotics for postoperative complications.

No statistically significant evidence was found for post-operative complications (Figure 4) following dental implant placement (*p* = 0.47, RR: 0.74; 95% CI 0.34–1.65). A risk ratio of 0.74 (95% CI 0.34–1.65), implies that antibiotics probably reduce the risk of post-operative complications by 26% (100 × (1 − RR)%).

## 4. Discussion

### 4.1. Dental Extraction

In general, few statistically significant results were seen across all included extraction studies for all the listed outcomes. Two out of the four extraction studies were regarded as low risk of bias [35,36,37], both of which reported that preoperative antibiotics were associated with reduced prevalence of bacteraemia levels and reduced risk of infection. Amoxicillin given preoperatively showed higher efficacy than moxifloxacin and clindamycin (*p* < 0.001 and *p* < 0.05 respectively). Amoxicillin was more favourable in reducing serum bacteraemia levels in comparison to placebo (*p* < 0.0001) [33]. Lacasa et al. (2007) found a statistically significant linear correlation between an increase in length of procedure and incidence of infection (*p* < 0.027). Dental extraction performed with ostectomy (bone removal) takes longer to perform and so this was evident in all arms of the trial vs. rate of infection without ostectomy.

Adverse events were reported in only two studies [35,37], of which only one study saw events occur in the placebo/ no antibiotic group. Only two minor adverse events had been reported in antibiotic groups for included studies (diarrhoea and itching). This indicates that these antibiotic regimens seem to have been well tolerated but due to the small sample size of patients included in this review it is not possible to assess the occurrence of other rare adverse events associated with antibiotic use such as anaphylactic shock.

There is a minor trend showing adverse events occurring more so in the antibiotic group across the only two trials which reported any adverse events (2.43% in antibiotic group vs. 0.71% in placebo group). However, results of the present review show there is no statistical significant results for adverse events between groups (*p* = 0.30). Based on these results, a risk ratio of 1.84 (95% CI 0.59–5.77) implies a patient is 84% more likely to experience adverse events with treatment than adverse events without treatment.

Overall, 2 out of 3 extraction studies [12,35] which report postoperative inflammatory complications as an outcome measure do not support the use of prophylactic antibiotics after third molar extraction (*n* = 237). Lacasa et al. (2006) found pre-emptive antibiotics are more beneficial than prophylactic antibiotics for complex extractive surgery requiring ostectomy (*p* = 0.006). The authors recommend single dose prophylaxis in simpler extractive procedures where ostectomy is not performed to reduce postoperative complications. Two of the lead authors of this phase III clinical study are employees of the drug company funding the phase III trial.

### 4.2. Dental Implants

Using three extraction studies (*n* = 711), the present study has found no statistically significant evidence to support the use of routine prophylactic antibiotics in reducing the risk of implant failure (*p* = 0.09, RR 0.43; 95% CI 0.16 to 1.14) and post-operative complications (*p* = 0.47, RR: 0.74; 95% CI 0.34–1.65). The only trial which used 3 various prophylactic antibiotic durations [14] failed to reveal conclusive evidence as not a single outcome event occurred in any arm of the trial (postoperative infection, prosthesis/implant failure, adverse events).

A risk ratio of 0.43 for implant failure implies that prophylactic antibiotics probably reduce the risk of implant failure by 57% based on these 711 patients under normal conditions. Antibiotics probably reduce the risk of post-operative complications following implant placement by 26% (RR: 0.74; 95% CI 0.34–1.65). Approximately 33 patients undergoing dental implant surgery need to receive antibiotics in order to prevent one implant failure from occurring (NNT, Table 7). This will cost £120.00 for 33 patients to receive a 2 g amoxicillin prophylactic dose [22] in order to prevent one implant failure. Although this may seem financially feasible, clinicians must carefully consider the increase in rate of antibiotic resistance and the chance of allergic/toxic reactions occurring before deciding to treat 33 patients in order to prevent one implant failure from occurring.

### 4.3. Other Views

A systematic review conducted by Esposito et al. (2013) assessed the beneficial or harmful effects of systemic prophylactic antibiotics at dental implant placement vs. placebo. Six randomised controlled clinical trials (RCTs) with a follow-up of at least three months were analysed which compared the administration of various prophylactic antibiotic regimens vs. placebo to patients undergoing dental implant placement. The authors conclude their evidence suggests that, in general, antibiotics are beneficial for reducing the failure of dental implants placed in ordinary conditions vs. placebo (*p* = 0.002, OR 0.33; 95% CI 0.19–1.00). No statistically significant differences were seen between groups regarding postoperative complications (*p* = 0.28, RR 0.60: 95% CI 0.36–1.35) or adverse events (*p* = 1.0, RR 1.0; 95% CI 0.006–15.85). The authors remain unsure whether postoperative antibiotics at dental implant are beneficial, and which antibiotic would be the most effective.

Schwartz & Larson (2007) conducted a detailed literature review to assess ‘antibiotic prophylaxis and postoperative complications following tooth extraction and implant placement’. Eight randomised clinical trials, one retrospective study and four additional randomised interventions were studied. In general sample sizes were small and provided insufficient statistical power to avoid type II, or false negative errors. The work emphasises how methodological differences in many of the dental clinical trials pose a problem regarding quality of evidence. The study concludes that there is a lack of evidence regarding the use of prophylactic antibiotics in general dentistry, including tooth extraction and implant placement [10].

A recent Cochrane systematic review conducted by Lodi et al. (2012) investigated the benefits of antibiotic prophylaxis in patients undergoing tooth extraction(s) for any indication. The authors found statistically significant evidence for a reduction in dry socket, pain, fever and trismus (*p* < 0.05). An increase in mild and transient adverse effects was observed in antibiotic groups vs. placebo. They conclude there is evidence that antibiotic prophylaxis reduces the risk of dry socket pain and infection following third molar extractive surgery. The main limitation of the review was almost half of the assessed trials were conducted before 1992 (6 of which were conducted in the 1980’s) and many of which used selective reporting, incomplete outcome data and wide variations in methodological approaches. All studies included were either high risk of bias or had an unclear risk of bias, and therefore this may question the reliability of results [38,39].

Martin et al. (2005) assessed the appropriateness of antibiotic prophylaxis for third molar extractive surgery. The body of evidence examined showed that antibiotics may provide certain benefits in certain circumstances and little or no benefit in other circumstances. For example, it was found prophylactic antibiotics may be beneficial in extractive surgery requiring bone removal. Despite this, the authors believe the body of evidence questions the benefit of routine prophylactic antimicrobial therapy which does not appear to overcome risk of undesirable outcomes after dental extraction of third molars [9].

### 4.4. Limitations

One of the main limitations of the present review is that only seven studies were identified and available for review. Although the clinical extraction studies used in this review were conducted in various countries worldwide, many of the patients were young healthy patients in their early twenties and so the results of the review were more applicable to healthy young adults undergoing surgical tooth extraction. In contrast, older participants were recruited across all included implant studies (varying between 42–52 years). No trials were identified which included young children, elderly patients or immune compromised patients requiring dental extractions, therefore the results of this review may not be applicable to this group although they would be expected benefit more from prophylactic antibiotics due to increased risk of infection. Indeed, the NNT for outcomes would be likely to decrease if this group of patients were to be included, however it may not be possible or ethical to conduct clinical studies using this group of patients. Extraction studies identified involved patients being treated by oral surgery specialists by referral and so again, it is unclear whether these results are relatable to general dental practice. No clinical extraction studies were found evaluating the use of prophylactic antibiotics for patients with periodontal complications or severe caries as these are the most common indications for dental extractive surgery of third molars.

### 4.5. Implications for Dentists

A clinician’s awareness about correct antibiotic choice is key to reducing ‘blind prescribing’, a factor which has contributed to the increase in antibiotic resistant microorganisms. Changes required in antibiotic prescribing habits presents its own set of problems as described by Soheilipour et al. (2011) whereby a qualitative study regarding the views of healthcare professionals on NICE guidelines revealed that prescribers experienced difficulty in explaining to their patients the change in clinical practice necessitated by adherence to the NICE guidance [40]. Concerns were also raised about the legal position of a clinician who did not follow the guidance. Further monitoring of antibiotic prescriptions among dentists is needed in order to effectively audit this controversial therapy. It is also recommended for continuing education of practitioners regarding the growing public health risks related to antibiotic prescriptions.

### 4.6. Implications for Further Work

There are varying opinions regarding the prophylactic use of antibiotics in dentistry [8,9,10]. More large scale randomised, double blind clinical studies need to be conducted. There is also a lack of clinical studies which have evaluated and defined the most appropriate and effective antibiotic regimen for dental procedures and so further work is recommended based on these proposals.

## 5. Conclusions

No statistically significant evidence was found to support the *routine* use of prophylactic antibiotics in reducing the risk of implant failure or post-operative complications under normal conditions. Approximately 33 patients undergoing dental implant surgery need to receive antibiotics in order to prevent one implant failure from occurring. Prophylactic antibiotics probably reduce the risk of implant failure by 57% based on 711 patients under normal conditions. There is also little conclusive evidence favouring the routine use of prophylactic antibiotics for third molar extractive surgery requiring bone removal in healthy young adults.

No trials were identified with the group of patients that would most likely benefit from the use of prophylactic antibiotics; elderly, young and immunocompromised patients. The results of the present study may therefore not be applicable to this group of patients. No trials were identified for the most common indications for dental extraction; dental caries or periodontal disease.

Much remains to be achieved in dental research including further large scale randomised, double-blind clinical studies using patients with infective complications such as infective endocarditis or immuno-compromised patients for various dental procedures.

Based on the articles analysed in this review it is recommended that clinicians carefully consider the appropriate use of antibiotics in dental implants and extraction procedures even if it is financially feasible due to risk of allergic/toxic reactions and the development of antibiotic resistance. Further monitoring of antibiotic prescribing in dentistry is required in addition to continuing education for dentists concerning the public health risks associated with antibiotic misuse.

## Figures and Tables

**Table 1:** Summary of commonly prescribed antimicrobial drugs in dentistry in the UK. Adapted from Ramu & Padmanabhan (2012) and the British National Formulary (BNF, 2013) [21,22].

Antibiotic	Class	Drug Mechanism	Spectrum of Activity	Common Indications in Dentistry	Dose Range	Comments
Amoxicillin	Penicillin	Inhibits biosynthesis of cell wall	Broad spectrum. Active against certain gram + and gram − organisms	Dentoavleolar abscess	250 mg three times daily (dose can be doubled in severe infections)	Just as effective as phenoxymethyl penicillin but better absorbed. Ineffective to beta lactamase producing organisms.
Ampicillin	Penicillin	Inhibits biosynthesis of cell wall	Broad spectrum. Active against certain gram + and gram − organisms	Dentoavleolar abscess	500–1000 mg four times daily	See amoxicillin
Penicillin V Phenoxymethylpenicillin	Penicillin	Inhibits biosynthesis of cell wall	More active against gram + than gram -	Dentoavleolar abscess. Should not be used in serious infections.	500 mg four times daily (dose can be doubled in severe infections)	Gastric acid-stable therefore suitable for oral administration unlike penicillin G
Co-Amoxiclav	Penicillin	Inhibits biosynthesis of cell wall	Broad spectrum. Active against beta-lactamase producing bacteria resistant to amoxicillin	Severe dental infection with spreading cellulitis or dental infection non-responsive to 1^st^ line antibacterial.	250 mg/125 mg (ampicillin/clauvic acid) combination tablet three times daily (higher dose of 500 mg/125 mg in severe infections)	A mixture of clauvulanic acid acting as beta-lacamase inhibitor (as potassium clavulanate) and amoxicillin (as trihydrate/sodium salt)
Cefalexin	Cephalosporin	Binds to penicillin binding proteins and inhibits cell wall synthesis.	More active against aerobes	Dental infections resistant to penicillin VK	250–1500 mg four times daily	Offer little advantage over penicillin’s in dental infections but useful in those with hypersensitivity to penicillin’s
Cefradine	Cephalosporin	Binds to penicillin binding proteins and inhibits cell wall synthesis.	More active against aerobes	Dental infections resistant to penicillin VK	250–1000 mg four times daily	See Cephalexin
Metronidazole	Metronidazole	Inhibiting nucleic acid synthesis	High activity against anaerobic bacteria and protozoa	Acute necrotising gingivitis, pericoronitis	200–250 mg three times daily	High concentration builds up achievable in tissue.
Clarithromycin	Macrolide	Inhibits bacterial peptide translation	Similar but not identical to penicillin	2^nd^ line drug treatment for dental abscess	250–500 mg twice daily	Many organisms rapidly develop resistance to macrolides; use should be limited to short courses
Doxycycline	Tetracycline	Inhibits bacterial protein synthesis	Effective against oral anaerobes.	Sinusitis	200 mg initially, 100 mg daily	Due to antibiotic resistance, especially by oral streptococci, tetracycline usefulness is reduced in acute oral infections.

* Local formulary dose recommendation may differ to BNF doses.

**Table 2:** Rationale for each level of bias.

Level of Bias	Description
High risk	Possible bias seriously affecting the reliability of the results and high risk of bias if one or more of the criteria were not met
Low risk	Possible bias not seriously affecting the reliability of the results and low risk of bias if all criterion met

**Table 3:** Risk of bias for studies based on 4 main criteria recommend by Cochrane Handbook for systematic reviews of interventions Version 5.1.0.

Study	Patient Blinding	Assessor Blinding	Allocation Concealment	Withdrawals	Risk of Bias
Anitua et al. (2009)	Yes	Yes	Concealed	None	Low
Caiazzo et al. (2011)	Yes	Yes	Unclear	None	High
Esposito et al. (2010)	Yes	Yes	Concealed	Yes (enough reasons have been provided)	Low
Sekhar et al. (2001)	Yes	Yes	Concealed	Yes	High
Dios et al. (2006)	Yes	Yes	Concealed	None	Low
Kaczmarzyk et al. (2007)	Yes	Yes	Concealed	Yes	High
Lacasa et al. (2007)	Yes	Yes	Unclear risk	None	Low

**Table 4:** A summary of study population characteristics.

Study (Author)	Extraction/Implant	Intervention	Number of Participants	Gender (m/f)	Mean Age (Years)	Number of Extractions/Implants
Caiazzo et al.	Implant	Amoxicillin 2 g 1 h pre-op	25	13/12	52	35
		Amoxicillin 2 g 1 h pre-op + amoxicillin 2 g daily for 7 days post-op	25	12/13	45	36
		Amoxicillin 2 g daily post-op for 7 days	25	7/18	42	48
		No antibiotic	25	10/15	43	29
Anitua et al.	Implant	Amoxicillin 2 g 1 h pre-op	52	15/37	49	52
		Placebo (identical tablests) 2 g 1 h pre-op	53	20/33	47	53
Esposito et al.	Implant	Amoxicillin 2 g 1 h pre-op	252	114/138	49.1	489
		Placebo (no antibiotic)	254	122/132	47.6	483
Sekhar et al.	Extraction	Metronidazole 1 g orally 1 h preoperatively	44	25/19	28	99
		Metronidazole 400 mg orally 4 times daily for 5 days	47	30/17	29	101
		placebo	34	15/19	26	103
Dios et al.	Extraction	Amoxicillin 2 g preoperatively	56	34/22	23.8	56
		moxifloxacin 400 mg preoperatively	58	29/29	22.4	58
		clindamycin 600 mg preoperatively	54	34/20	24	54
		Placebo	53	29/24	26.1	53
Kaczmarzyk et al.	Extraction	Clindamycin 600 mg preoperatively then 300 mg placebo for 5 days	31	8/23	23.4	31
		Clindamycin 600 mg preoperatively then 300 mg placebo for 5 days post-op	28	9/19	23.5	28
		placebo	27	6/21	24.6	27
Lacasa et al.	Extraction	Pre-op amoxicillin/clavulanate 2000/125 mg	75	33/42	29.7	75
		post-op amoxicillin/clavulanate 2000/125 mg	75	37/38	29.5	75
		placebo	75	26/49	28.2	75

**Table 5:** A summary of key characteristics for randomised clinical extraction studies evaluating the effectiveness of antibiotics in preventing post-operative complications.

Study (Author)	Complication/Procedure	Intervention	Patient Sample Size	Study Design	Outcomes Assessed	Location	Results	Comments
Sekhar et al. (2001)	Lower wisdom tooth extraction	Metronidazole 1 g orally 1h preoperatively vs. metronidazole 400 mg orally 4 times daily for 5 days vs placebo	*n* = 151	3-arm, randomised, double blind	Purulent discharge from wound, dry socket, swelling, pain score	India	Overall, no significant differences in groups from any of the variables.	Outcome assessment procedures were not clearly specified. No power analysis performed. At enrolment patients’ key characteristics not fully assessed.
Kaczmarzyk et al. (2007)	Extraction of third molar tooth.	Clindamycin 600 mg preoperatively then 300 mg placebo vs. Clindamycin 600 mg preoperatively then 300 mg placebo post-op vs. placebo (5 day treatment)	*n* = 86	3-arm prospective, randomised, double blind	Using 4-grade scale: Trismus, facial swelling, pain, body temperature & alveolar osteitis. All evaluated on day 1, 2 and 7 (post-op)	Poland	No statistically significant differences in post-op complication rates for third molar extraction from any group.	Incomplete outcome data (attrition bias)—14% patients lost at follow up. Inclusion criteria basic. Exclusion criteria well described. Power analysis performed. Demographic, objective and subjective data clearly defined.
Dios et al. (2006)	Tooth extraction for any indication	Amoxicillin 2 g preoperatively vs. moxifloxacin 400 mg vs. clindamycin 600mg (preoperatively vs. placebo (5 day treatment)	*n* = 221	3-arm, randomised, double blind	Postoperative bacteraemia levels determined by microbiological analysis of blood cultures.	Spain	Postoperative measurements of bacteraemia showed decrease in amoxicillin and moxifloxacin (*p* < 0.0001) vs. placebo	Clear exclusion criteria described, however unclear description of inclusion criteria. Power analysis performed.
Lacasa et al. (2006)	Third mandibular surgery required	Pre-op amoxicillin/clavulanate 2000/125 mg vs. post-op amoxicillin/clavulanate 2000/125 mg vs. placebo (5 day treatment)	*n* = 225	3-arm randomised, double blind, parallel, phase III comparative study	Infection (purulent discharge in surgical site, pain, local abscess, increased heat, pyrexia, trismus, dental osteitis. All evaluated on days 1,3,7 post-op.	Spain	Higher rate of infection was seen in placebo group (16%) vs. single dose prophylaxis (5.3%) vs. 5 day pre-emptive therapy (2.7%) (*p* = 0.006)	Patients lost at each follow up not mentioned. Incomplete outcome data (attrition bias). Randomisation method not clearly defined. Two authors are employed by the funding company.

**Table 6:** A summary of key characteristics for randomised clinical implant studies evaluating the effectiveness of antibiotics in preventing implant failure.

Study (Author)	Complication/Procedure	Intervention	Sample Size	Study Design	Outcomes Assessed	Location	Results	Comments
Caiazzo et al. (2010)	Dental implant surgery	Amoxicillin 2 g pre-op vs. Amoxicillin 2 g daily pre & post-op (7days) vs. amoxicillin 2 g post-op (7days) vs. placebo	*n* = 100	4-arm, prospective, multicentre parallel, randomised, study (3 month study)	Implant failure, postoperative complications assessed post-op at weeks 1,2,4 and 8, adverse events	Italy	Overall success rate 98.65%. No significant differences between expt. groups (*p* < 0.05). no implant failures in antibiotic groups, 2 failures in non-antibiotics groups.	Allocation concealment information not provided. No information provided for blinding of operators.
Esposito et al. (2010)	Dental implant surgery	Amoxicillin 2g pre-op vs. placebo	*n* = 506	Randomised, multicentre, double blind, placebo controlled & parallel (4 month duration)	Implant & prosthesis failure. Post-op complications (assessed weeks 1 & 2 post-op), and adverse events.	Italy	No statistically significant differences observed between groups but trend favoured antibiotic administration. More implant losses in placebo group (*p* = 0.083)	Limitations of the study well described. Allocation concealment information well provided. Information provided for blinding of operators. All outcome measures reported.
Anitua et al. (2009)	Dental implant surgery	Amoxicillin 2g preoperatively vs. moxifloxacin 400 mg vs clindamycin 600 mg preoperatively vs placebo	*n* = 105	Randomised, multicentre, double blind, parallel, placebo controlled	Postoperative infections, microbiological analysis, adverse events and implant failures.	Spain	Six post op infections occurred and 2 implant failures in each group. No statistically significant differences observed between groups.	No patient drop outs. Results may be applicable to bone types II & 3 only. No other bone types investigated.

**Table 7:** Number need to treat calculation.

Numbers Need to Treat (NNT)
Control group event rate (CER): proportion of outcomes that occur in control group. Experimental group event rate (EER): proportion of outcomes that occur in the experimental group. Absolute risk reduction (ARR) = CER − EER Number needed to treat (NNT) = 1/ARR To prevent implant failure using prophylactic antibiotics: CER = 16/332 = 0.048 EER = 7/379 = 0.018 Absolute risk reduction (ARR) = 0.048 − 0.018 = 0.030 NNT = 1/0.030 = 33.333 Approximately 33 patients undergoing dental implant surgery need to receive antibiotics in order to prevent one implant failure from occurring (NNT)

## Appendix A. Outcome Measures and Statistical Analysis Using REVMAN 5.0 Software

Data was pooled using REVMAN 5.0 software. The relative risk (RR) with 95% confidence intervals (CIs) for dichotomous data (data with two mutually exclusive groups, i.e., occurrence of implant failure or not, presence or absence of adverse events and presence and absence of postoperative complications was calculated for each study to quantitatively measure the probability of an event occurring. Where possible, based on the reviewed study design, the numbers needed to treat (NNT) were calculated to measure the number of patients who need to be treated to prevent one additional adverse outcome.

## Appendix B. CASP Forms Checklists Templates Were Used from CASP International Network (2013)

**CASP Analysis of Dios et al. (2006)**

**Section (A) Are the results of the review valid?**
**Screening Questions** **1. Did the trial address a clearly focused issue?**
Yes. The authors evaluated the efficacy of Amoxicillin 2 g preoperatively vs moxifloxacin 400 mg vs. clindamycin 600 mg preoperatively vs. placebo (5-day treatment) for the prevention of bacteraemia following dental extraction.
**2. Was the assignment of patients to treatments randomised?**
Yes. Randomisation was based on a single sequence of random assignments using a computer generated randomisation list.
**3. Were all of the patients who entered the trial properly accounted for at its conclusion?**
Yes. All 221 patients had been accounted for in results and conclusion of the study
**Detailed questions** **4. Were patients, health workers and study personnel ‘blind’ to treatment?**
Study is described as ‘double-blind’.
**5. Were the groups similar at the start of the trial?**
**Size and composition of all three groups were similar** Amoxicillin 2 g preoperatively (*n* = 56): 34 (M)/22 (F), mean age = 23.8 years Moxifloxacin 400 mg preoperatively (*n* = 58): 29 (M)/29 (F), mean age = 22.4 years Clindamycin 600 mg preoperatively (*n* = 54): 34 (M)/20 (F), mean age = 24 years Placebo (*n* = 53): 29 (M)/24 (F), mean age = 26.1 years The range of total extractions per group ranged from 53–58.
**6. Aside from the experimental intervention were the groups treated equally?**
All patients were subjected to the same inclusion/ exclusion criteria and all extractions were conducted in the Santiago de Compostela University Hospital (Spain) under general anaesthesia.
**Section (B) What are the results?**
**7. How large was the treatment effect?**
The presence of bacteraemia following dental extraction was measured as the outcome of interest. *Streptococcu*s spp. were the most commonly identified bacteria in all groups ranging from 44 to 68% with the lowest percentage being detected from the amoxicillin group (*p* < 0.0001). Amoxicillin and moxifloxacin prophylaxis showed high efficacies (*p* < 0.001 and *p* < 0.05 respectively) in reducing prevalence and duration or bacteraemia. Clindamycin prophylaxis was seen to be non-effective (*p* < 0.9).
**8. How precise was the estimate of the treatment effect?**
No confidence intervals are provided in the study, however *p*-values have been provided.
**Section (C) Will the results help locally?**
**9. Can the results be applied in your context?** **(or to the local population?)**
Results may not be applicable to a general population. Patients did not comprise of the elderly, young children, immunocompromised patients or those at risk of IE. Patient mean age was in mid-20’s. A larger sample size would be required. Furthermore, the procedures were carried out in a hospital setting rather than a dental practice. General anaesthesia was used, but local anaesthetic is more commonly for tooth extraction (depending on complexity of extraction).
**10. Were all clinically important outcomes considered?**
No. Adverse events were not considered as an outcome measure. Signs of infection were not considered as outcome measures.
**11. Are the benefits worth the harms and costs?**
The study shows that amoxicillin and moxifloxacin prophylaxis showed high efficacies (*p* < 0.001 and *p* < 0.05 respectively) in reducing prevalence and duration or bacteraemia following dental extraction, and therefore beneficial. This of course in turn would be highly likely to reduce postoperative complications. Commonly prescribed antibiotics used in dentistry are relatively cheap in the UK, however the type of antibiotic prescribed to patients should be strongly considered by prescribers as well as considering the genuine need for that particular patient, for example, in general, the elderly, immunocompromised and younger patients would seem more likely to benefit from prophylactic antibiotics as opposed to blind prescribing for each and every individual undergoing tooth extraction.

**CASP Analysis of Sekhar et al. (2001)**

**Section (A) Are the results of the review valid?**
**Screening Questions** **1. Did the trial address a clearly focused issue?**
Yes. The authors attempted to test the efficacy of two dosing regimens of prophylactic antibiotics for the removal of impacted third molars. The Intervention used was metronidazole 1 g orally 1hr preoperatively vs. metronidazole 400 mg orally 4 times daily for 5 days vs. placebo.
**2. Was the assignment of patients to treatments randomised?**
Method of sequence generation was not fully described; however, study claims to have used randomisation in sealed envelopes (allocation).
**3. Were all of the patients who entered the trial properly accounted for at its conclusion?**
151 patients were randomised at the beginning of the study and 125 were accounted for at its conclusion. The results of those who did not complete the study were also provided and statistically analysed, finding no significant differences between groups.
**Detailed questions** **4. Were patients, health workers and study personnel ‘blind’ to treatment?**
The outcome assessor was blinded to allocated treatment. Study is described as double-blind (dosing schedule is different in each group).
**5. Were the groups similar at the start of the trial?**
**Size of all four groups saw slight variation in number of participants shown below. The composition of each group saw slight variations:** Metronidazole 1 g orally 1 h preoperatively (*n* = 44): 25 (M)/19 (F), mean age = 28 years Metronidazole 400 mg orally 4 times daily for 5 days (*n* = 47): 30 (M)/17 (F), mean age = 29 years Placebo (*n* = 34): 15 (M)/19 (F), mean age = 26 years The range of total extractions per group ranged from 99–103.
**6. Aside from the experimental intervention were the groups treated equally?**
All patients were subjected to the same inclusion/exclusion criteria and all extractions were conducted in one treatment centre in India. It is worthy to note however that surgeons performing the extraction were of different skill levels i.e., some were consultants, post-graduate trainees or house officers.
**Section (B) What are the results?**
**7. How large was the treatment effect?**
No significant differences in outcome between three groups (*p* = 0.09). The only significant difference seen was the degree of swelling in the 5 day group (*p* = 0.03). Overall, antibiotic prophylaxis does not seem to reduce morbidity after third molar extraction surgery.
**8. How precise was the estimate of the treatment effect?**
No confidence intervals are provided. Only standard deviation figures were provided for individual results. Overall the study showed no statistically significant results to recommend prophylactic antibiotics for the extraction of third molars.
**Section (C) Will the results help locally?**
**9. Can the results be applied in your context?** **(or to the local population?)**
Results may not be applicable to a general population. Patients did not comprise of the elderly, young children, immunocompromised patients or those at risk of IE. A larger sample size would be required. Furthermore, surgeons were of different skill levels and the procedure itself was not carried out in a dental practice were most extractions are carried out in the UK.
**10. Were all clinically important outcomes considered?**
No. Adverse events were not considered as an outcome measure.
**11. Are the benefits worth the harms and costs?**
Results were not statistically significant (*p* > 0.05), and so there is not enough evidence to support the use of antibiotics in this case. Metronidazole is relatively cheap in the UK but the risk of other complications such as microbial resistance may outweigh the benefit if it is to be routinely prescribed for a procedure which in this case has failed to show statistically significant results.

**CASP Analysis of Esposito et al. (2010)**

**Section (A) Are the results of the review valid?**
**Screening Questions** **1. Did the trial address a clearly focused issue?**
Yes. To evaluate the efficacy of antibiotic prophylaxis for dental implant placement. The intervention used was amoxicillin 2 g pre-op vs. amoxicillin 2 g daily pre & post-op (7 days) vs. amoxicillin 2 g post-op (7 days) vs. placebo. Outcomes considered were implant failure, postoperative complications assessed post-op at weeks 1, 2, 4 and 8, adverse events.
**2. Was the assignment of patients to treatments randomised?**
Yes. Computer generated restricted randomisation lists (13 in total) were formed using equal groups of participants. Randomised codes were enclosed in sequentially numbered, identical, opaque, sealed envelopes. Treatment allocation was concealed to investigators in charge of enrolling and treating the patients.
**3. Were all of the patients who entered the trial properly accounted for at its conclusion?**
Two exclusions were made from the control group (antibiotic group) and one from the non-antibiotic group with reasons provided. Patients were analysed in groups they were randomised to.
**Detailed questions** **4. Were patients, health workers and study personnel ‘blind’ to treatment?**
Yes. Patients and operators/outcome assessors were blinded to intervention. The statistician was also kept blind when performing all analyses for this study
**5. Were the groups similar at the start of the trial?**
Yes. Similar Amoxicillin group (*n* = 52): 114 (M)/138 (F). Mean age 49.1 Placebo group (*n* = 254): 112 (M)/132 (F). Mean age 47.9
**6. Aside from the experimental intervention were the groups treated equally?**
Yes. Patients were treated in 10 private Italian practices. The number of implant placements for each group were similar (498; antibiotic group vs. 483; control group).
**Section (B) What are the results?**
**7. How large was the treatment effect?**
No statistically significant differences were seen to be observed. However trends to favour the antibiotic group. The study furthermore concludes immediate post-extractive implants were more likely to fail. Implant failure: 7 in antibiotic group vs. 13 placebo group Prosthesis failure: 4 failures in antibiotic group vs. 10 placebo group Adverse events: none reported. Post-operative complications: 11 in antibiotic group vs. 13 in placebo group
**8. How precise was the estimate of the treatment effect?**
Confidence interval not provided. *p* = 0.083 for overall implant losses (not statistically significant).
**Section (C) Will the results help locally?**
**9. Can the results be applied in your context?** **(or to the local population?)**
Cannot tell. Implant placement is not limited to sex/age. Most patients in this trial were >40 years. Results may not be generalisable to a whole general population. Patients did not comprise of the elderly, young children, immunocompromised patients or those at risk of IE. A larger sample size would be required.
**10. Were all clinically important outcomes considered?**
Yes. All outcomes considered and reported, however confidence intervals should have been provided. A different dosing regimen group could also have been used.
**11. Are the benefits worth the harms and costs?**
Can’t tell. Trends are clearly in favour of antibiotics as more implant, prosthesis failures and post-operative complications were seen in the placebo group vs. antibiotic group. Despite this the results were not statistically significant (*p* = 0.083). No adverse events were reported but this may be due to the small sample size of the study. Amoxicillin is relatively cheap in the UK but the risk of other complications such as microbial resistance may outweigh the benefit if it is to be routinely prescribed for a procedure which fails to show statistically significant results.

**CASP Analysis of Kaczmarzyk et al. (2007)**

**Section (A) Are the results of the review valid?**
**Screening Questions** **1. Did the trial address a clearly focused issue?**
Yes. The authors attempted to evaluate the efficacy of two antibiotic regimens (single dose clindamycin and multidose clindamycin (5 days) for the reduction of inflammatory complications in patients undergoing third molar surgery with bone removal (extraction). Outcomes of interest were trismus, facial swelling, body temperature, pain, submandibular lynphadenopathy and alveolar osteitis.
**2. Was the assignment of patients to treatments randomised?**
A random number table was used for to determine group assignment for each patient in advance. Furthermore, allocation concealment involved opaque and sequentially numbered envelopes.
**3. Were all of the patients who entered the trial properly accounted for at its conclusion?**
Nine out of 100 patients did not check in for follow up. Three had been disqualified due to various described complications and two had resigned during the trail without stating any reasons. Therefore 86 patients in total were analysed statistically at the end of the trial.
**Detailed questions** **4. Were patients, health workers and study personnel ‘blind’ to treatment?**
Patients, clinicians and statisticians were all blinded.
**5. Were the groups similar at the start of the trial?**
Sizes of all three groups were similar (*n* = 27–31) The composition of each group was very similar except one group composed of a higher female population. Clindamycin 600 mg preoperatively then 300 mg placebo for 5 days: 8 (M)/23 (F), mean age = 23.4 years Clindamycin 600 mg preoperatively then 300 mg placebo for 5 days post-op: 9 (M)/19 (F), mean age = 23.5 years placebo: 6 (M)/21 (F), mean age = 24.6 years Total extractions between groups ranged from 27–31.
**6. Aside from the experimental intervention were the groups treated equally?**
Yes. All groups were treated at one treatment center in Poland and subject to the same inclusion/exclusion criteria.
**Section (B) What are the results?**
**7. How large was the treatment effect?**
There was no statistically significant differences observed between groups for postoperative alveolar osteitis, pain scores, postoperative trismus and facial swelling (*p* > 0.05). Regarding a change in body temperature, a statistically significant difference between groups was recorded (*p* = 0.03). Despite this one statistically significant result for this study, the authors conclude that there is not enough evidence to suggest that clindamycin used prophylactically by itself or with subsequent 5-day therapy fails to demonstrate significant efficacy for prevention of inflammatory complications after third molar surgery.
**8. How precise was the estimate of the treatment effect?**
The *X*^2^ test was used to evaluate trismus, facial swelling, lymphadenopathy and alveolar osteitis using an odds ratio with a confidence interval of 95%. The Krukal-Wallis rank test was used to analyse surgery duration, analgesic intake, body temperature and level of pain experienced.
**Section (C) Will the results help locally?**
**9. Can the results be applied in your context?** **(or to the local population?)**
Results may not be applied to a whole general population. Patients were not treated in a general dental surgery, where most extractions take place. Patients did not comprise of the elderly, young children, immunocompromised patients or those at risk of IE. The mean age of patients across all three groups was in the mid 20’s, and so they may be more applicable to younger adults, however even then, larger sample sizes than this study would also require to represent a typical population of patients.
**10. Were all clinically important outcomes considered?**
No. All-important outcomes were considered except adverse events were not reported.
**11. Are the benefits worth the harms and costs?**
Results described above are not statistically significant. In the case of this study, adverse events were not reported. With regards to cost, clindamycin is relatively cheap in the UK but the risk of other complications such as microbial resistance may outweigh the benefit if it is to be routinely prescribed for an extraction procedure which is uncomplicated and of short duration. Overall, the study shows there is little benefit from taking prophylactic antibiotics for the reduction in inflammatory complications following third molar surgery.

**CASP Analysis of Lacasa et al. (2007)**

**Section (A) Are the results of the review valid?**
**Screening Questions** **1. Did the trial address a clearly focused issue?**
Yes. The authors attempted to evaluate the efficacy of two sustained release antibiotic regimens for the reduction of infection after third molar surgery (extraction): Pre-op amoxicillin/clavulanate 2000/125 mg vs post-op amoxicillin/clavulanate 2000/125 mg vs. placebo (5 day treatment). Outcomes of interest were Infection (purulent discharge in surgical site, pain, local abscess, increased heat, pyrexia, trismus, dental osteitis. All evaluated on days 1, 3, 7 post-op.
**2. Was the assignment of patients to treatments randomised?**
Allocation concealment is not mentioned and therefore an unclear risk. Also random sequence generation is mentioned as randomised, however the method of how it was generated has not been provided.
**3. Were all of the patients who entered the trial properly accounted for at its conclusion?**
Every patient was accounted for at conclusion.
**Detailed questions** **4. Were patients, health workers and study personnel ‘blind’ to treatment?**
All were blinded.
**5. Were the groups similar at the start of the trial?**
Size of all four groups were identical (*n* = 75 for each group). The composition of each group was very similar except one group composed of a higher female population. Pre-op amoxicillin/clavulanate 2000/125 mg: 33 (M)/42 (F), mean age = 29.7 years post-op amoxicillin/clavulanate 2000/125 mg: 37 (M)/48 (F), mean age = 52 years placebo: 26 (M)/49 (F), mean age = 28.2 years Each group had a total of 75 extractions (1 extraction per patient)
**6. Aside from the experimental intervention were the groups treated equally?**
Yes. All groups were treated at one treatment centre and subject to the same inclusion/exclusion criteria.
**Section (B) What are the results?**
**7. How large was the treatment effect?**
Higher rate of infection was seen in placebo group (16%) vs. single dose prophylaxis (5.3%) vs. 5 day pre-emptive therapy (2.7%) (*p* = 0.006) meaning results were statistically significant. A significant result was seen in the correlation between the longer duration of surgery and rate of infection (*p* = 0.011) (although initially this was not an outcome of interest). Pre-emptive and pre-operative antibiotics vs. placebo saw a greater reduction of postoperative pain on day 3 (*p* = 0.0001) Overall pre-emptive therapy is better suited for more complex procedures (extraction including bone removal). Prophylaxis is more beneficial in simpler procedures.
**8. How precise was the estimate of the treatment effect?**
Details on CI were not included for results of the study; however they were provided for the reasoning behind why 75 patients were required in each study group in order to achieve a sufficient sample size (based on a previous pilot study with smaller samples sizes)
**Section (C) Will the results help locally?**
**9. Can the results be applied in your context?** **(or to the local population?)**
Results may not be generalisable to a whole general population. Patients were not treated in a general dental surgery, where most extractions take place. Patients did not comprise of the elderly, young children, immunocompromised patients or those at risk of IE. The mean age of patients across all three groups was in the late 20’s. A larger sample size would also be required.
**10. Were all clinically important outcomes considered?**
Yes, however the CI for each outcome considered was not provided (although *p* values were).
**11. Are the benefits worth the harms and costs?**
Results described above are statistically significant. Generally adverse events were seen more frequently in the pre-emptive antibiotic group and with greater effect compared to prophylaxis and placebo groups. Therefore the choice of antibiotic regimen should be carefully considered when judging the difficulty of extraction and the benefits of administration to each individual patient. With regards to cost, amoxicillin is relatively cheap in the UK but the risk of other complications such as microbial resistance may outweigh the benefit if it is to be routinely prescribed for an extraction procedure which is uncomplicated and of short duration.

**CASP Analysis of Anitua et al. (2010)**

**Section (A) Are the results of the review valid?**
**Screening Questions** **1. Did the trial address a clearly focused issue?**
Yes. The study compared the efficacyand safety of oral amoxicillin 2 g with identical placebo tablets taken prophylacticaly (1 h before implant placement) for single dental implants in bone type 2 & 3. Outcomes considered. Outcomes of interest included implant failure, postoperative complications assessed post-op at weeks 1, 2, 4 & 8, adverse events and microbiological evaluation.
**2. Was the assignment of patients to treatments randomised?**
How was this carried out? Was the allocation sequence concealed from researchers and patients? Yes. Researchers had a concealed envelope for each patient to establish randomly assigned treatment if necessary (envelope opened at end of study). If a side effect was observed, then the clinician was allowed to open the envelope before the end of the study.
**3. Were all of the patients who entered the trial properly accounted for at its conclusion?**
A 52 patients were enrolled into the antibiotic group and 53 patients were enrolled into the placebo group for which results for all patients were given at the end of the study.
**Detailed questions** **4. Were patients, health workers and study personnel ‘blind’ to treatment?**
Yes. Researchers and patients were blinded to the received treatment group.
**5. Were the groups similar at the start of the trial?**
Size of both groups were almost identical (antibiotic group *n* = 52 & non antibiotic group *n* = 53. The composition of each group was also similar except the male to female ratio in one of the groups: Amoxicillin 2 g 1h pre-op: 15 (M)/37 (F), mean age = 49 years. Placebo (identical tablets) 2 g 1hr pre-op: 20 (M)/33 (F), mean age 53.
**6. Aside from the experimental intervention were the groups treated equally?**
Yes. This study only included people requiring a single implant into bone of medium quality. Only one implant was provided to each patient in each group. Patients were also treated in same country (8 Spanish dental practices)
**Section (B) What are the results?**
**7. How large was the treatment effect?**
Outcome measures included: Implant failures: 2 failures occurred in each group Adverse events: none reported Postoperative infections: 6 in each group. The probability of not having and infection was 88.8% in the non-antibiotic group vs. 88.8% in the antibiotic group. No statistical differences reported (*p* = 0.0960) Characteristics of saprophytic flora: no statistically significant results observed, the amoxicillin did not alter or modify the nature of the saprophytic flora (*p* = 0.362) Overall the study concludes that there is no statistically significant data to suggest antibiotic prophylaxis when placing single implants in patients with bone types 2 & 3.
**8. How precise was the estimate of the treatment effect?**
The type of treatment applied did not significantly affect the probability of occurrence of infections (OR 0.97—CI 95% 0.29–3.2)
**Section (C) Will the results help locally?**
**9. Can the results be applied in your context?** **(or to the local population?)**
Results may not be generalisable to a whole general population. Patients did not comprise of the elderly, young children, immunocompromised patients or those at risk of IE. A larger sample size would be required. Furthermore, only patients with bone types 2 & 3 were used and so results are more applicable to these patients.
**10. Were all clinically important outcomes considered?**
Yes. All outcomes considered and reported. A different dosing regimen group could also have been tested.
**11. Are the benefits worth the harms and costs?**
Not in this case. Insufficient statistically significant evidence. Results were not statistically significant (*p* > 0.05). Amoxicillin is relatively cheap in the UK but the risk of other complications such as microbial resistance may outweigh the benefit if it is to be routinely prescribed for a procedure which fails to show statistically significant results.

**CASP Analysis of Caiazzo et al. (2010)**

**Section (A) Are the results of the review valid?**
**Screening Questions** **1. Did the trial address a clearly focused issue?**
Yes. The authors attempted to determine the minimum effective regimen of antibiotic prophylaxis (amoxicillin) for dental implant surgery. The intervention used was amoxicillin 2 g pre-op vs. amoxicillin 2 g daily pre-& post-op (7days) vs. amoxicillin 2 g post-op (7days) vs. placebo.
**2. Was the assignment of patients to treatments randomised?**
No information regarding the allocation concealment procedure was provided. A computer generated randomisation list was produced to allocate patients into one of four groups.
**3. Were all of the patients who entered the trial properly accounted for at its conclusion?**
All adults were treated in two private Italian dental practices. Results for all 100 patients (4 groups of 25) who entered the trial were provided at the end of the trial.
**Detailed questions** **4. Were patients, health workers and study personnel ‘blind’ to treatment?**
Operators were not blinded as they were recording the outcome measures.
**5. Were the groups similar at the start of the trial?**
Size of all four groups were identical (*n* = 25 for each group). The composition of each group saw slight variations: Amoxicillin 2 g 1 h pre-op (*n* = 25): 13 (M)/12 (F), mean age = 52 years Amoxicillin 2 g 1 h pre-op + amoxicillin 2 g daily for 7 days post-op (*n* = 25): 12 (M)/13 (F), mean age = 45 years Amoxicillin 2 g daily post-op for 7 days (*n* = 25): 7 (M)/18 (F), mean age = 42 years No antibiotic (*n* = 25): 10 (M)/15 (F), mean age = 43 years The range of total implants per group ranged from 29–48.
**6. Aside from the experimental intervention were the groups treated equally?**
Yes. All groups were treated at two Italian private dental clinics subject to the same inclusion/exclusion criteria.
**Section (B) What are the results?**
**7. How large was the treatment effect?**
Outcomes of interest included: Implant failure: 2 implant failures in no antibiotic group No postoperative complications observed post-op at weeks 1, 2, 4 and 8 Adverse events: none reported
**8. How precise was the estimate of the treatment effect?**
Power analysis showed only 15% with confidence at 99% and 35% at 95% CI. However further calculations revealed that in order to achieve a power of 75% with 99% confidence, 133 samples are required in each group; a much larger quantity than the current 25 per group is required to further provide strong statistical evidence of the absence of treatment effect.
**Section (C) Will the results help locally?**
**9. Can the results be applied in your context?** **(or to the local population?)**
Results may not be generalisable to a whole general population. Patients did not comprise of the elderly, young children, immunocompromised patients or those at risk of IE. A larger sample size would be required.
**10. Were all clinically important outcomes considered?**
Yes. All outcomes considered and reported. A different dosing regimen group could also have been tested.
**11. Are the benefits worth the harms and costs?**
Not in this case. Insufficient statistically significant evidence (*p* > 0.05). No adverse events were reported but this may be due to the small sample size of the study. Amoxicillin is relatively cheap in the UK but the risk of other complications such as microbial resistance may outweigh the benefit if it is to be routinely prescribed for a procedure which fails to show statistically significant results.
